# Infection biomarkers in primary care patients with acute respiratory tract infections–comparison of Procalcitonin and C-reactive protein

**DOI:** 10.1186/s12890-016-0206-4

**Published:** 2016-03-24

**Authors:** Marc Meili, Alexander Kutz, Matthias Briel, Mirjam Christ-Crain, Heiner C. Bucher, Beat Mueller, Philipp Schuetz

**Affiliations:** University Department of Medicine, Kantonsspital Aarau, Tellstrasse 5001, Aarau, Switzerland; Basel Institute for Clinical Epidemiology and Biostatistics, Department of Clinical Research, University Hospital Basel, Basel, Switzerland; Division of Endocrinology, Diabetology and Clinical Nutrition, University Hospital Basel, Basel, Switzerland; Department of Clinical Epidemiology and Biostatistics, McMaster University, Hamilton, Canada

**Keywords:** Procalcitonin, C-reactive protein, Acute respiratory tract infection, Primary care, Outcome

## Abstract

**Background:**

There is a lack of studies comparing the utility of C-reactive protein (CRP) with Procalcitonin (PCT) for the management of patients with acute respiratory tract infections (ARI) in primary care. Our aim was to study the correlation between these markers and to compare their predictive accuracy in regard to clinical outcome prediction.

**Methods:**

This is a secondary analysis using clinical and biomarker data of 458 primary care patients with pneumonic and non-pneumonic ARI. We used correlation statistics (spearman’s rank test) and multivariable regression models to assess association of markers with adverse outcome, namely days with restricted activities and persistence of discomfort from infection at day 14.

**Results:**

At baseline, CRP and PCT did not correlate well in the overall population (r^2^ = 0.16) and particularly in the subgroup of patients with non-pneumonic ARI (r^2^ = 0.08). Low correlation of biomarkers were also found when comparing cut-off ranges, day seven levels or changes from baseline to day seven. High baseline levels of CRP (>100 mg/dL, regression coefficient 1.6, 95 % CI 0.5 to 2.6, sociodemographic-adjusted model) as well as PCT (>0.5ug/L regression coefficient 2.0, 95 % CI 0.0 to 4.0, sociodemographic-adjusted model) were significantly associated with larger number of days with restricted activities. There were no associations of either biomarker with persistence of discomfort at day 14.

**Conclusions:**

CRP and PCT levels do not well correlate, but both have moderate prognostic accuracy in primary care patients with ARI to predict clinical outcomes. The low correlation between the two biomarkers calls for interventional research comparing these markers head to head in regard to their ability to guide antibiotic decisions.

**Trial registration:**

Current Controlled Trials, ISRCTN73182671

**Electronic supplementary material:**

The online version of this article (doi:10.1186/s12890-016-0206-4) contains supplementary material, which is available to authorized users.

## Background

Acute respiratory tract infections (ARIs) are the most common reason for antibiotic therapy in primary care [1, 2]. Yet, about 70 % of ARIs are treated with antibiotics, despite their mainly viral etiology [3]. As a consequence, antibiotic overuse results in increased antibiotic resistance, antibiotic-induced adverse events, and costs [4, 5]. A novel approach for more individualized and targeted antibiotic therapy is the use of blood biomarkers, such as C-reactive protein (CRP) or Procalcitonin (PCT) [6–8]. Several trials in primary care have shown that the use of one of these markers reduces antibiotic exposure, although head to head comparison trials are currently lacking [9–11]. A recent systematic search and meta-analysis found a total of four observational studies from primary care looking at CRP and PCT for their ability to predict pneumonic infiltrate, need for hospitalization, and diagnosis of group A *streptococcus* pharyngitis [9]. Also, previous observational studies compared the diagnostic performance of CRP and PCT in the hospital setting [12, 13] and in septic patients in the ICU setting [14, 15]. However, a comprehensive comparison analysis between these two markers in the primary care setting is currently lacking. Importantly, so far PCT protocols have been used mainly for management of hospitalized and intensive care patients due to the lack of high sensitive point of care tests (POCT). Recently, a sensitive POCT has been developed and may be increasingly used in primary care [16].

Herein, using data from a previous trial, our aim was to study how well CRP and PCT correlate, and their predictive value for outcome discrimination in a large-scale well-defined ARI cohort [17, 18].

## Methods

### Study design and setting

This predefined ancillary project included all patients with upper and lower ARI enrolled between December 2004 and April 2006 into the PARTI trial (Procalcitonin-guided antibiotic use versus a standard approach for acute respiratory tract infections in primary care) [17]. Study details have been reported elsewhere [18]. In brief, 53 primary care physicians in northwest of Switzerland consecutively screened adults with symptoms of an ARI and, in their physician’s opinion in need of antibiotics, were randomized to either a PCT-guided approach to antibiotic therapy or to a standard approach where physicians were asked to adhere to current guidelines. The primary non-inferiority outcome was the number of days, during the first 14 days after baseline, where a patient’s activities were restricted by an ARI.

### Selection of participants

All patients with the diagnosis of upper or lower RTI and the physician’s intention to prescribe antibiotics, based on evidence guidelines, were consecutively included. Exclusion criteria were antibiotic use within the previous 28 days, psychiatric disorders or inability to give written informed consent, not being available for follow-up, not being fluent in German, severe immunosuppression, cystic fibrosis, active tuberculosis, and the need for immediate hospitalization.

Classification of patients was standardized according to previously defined guidelines for the correct diagnosis of patients with ARI [18]. For these subgroup analyses we divided patients into three groups based on the initial evaluation by a primary care physician: *upper ARI* comprised patients with rhinosinusitis, pharyngitis/tonsillitis, and otitis media; *lower non*-*pneumonic ARI* comprised patients with common cold, tracheo-bronchitis, influenza, acute exacerbation of asthma or chronic obstructive pulmonary disease, and *lower pneumonic ARI* comprised patients with community-acquired pneumonia (CAP).

### Biomarker measurement

Blood samples were collected from all recruited patients on admission and day seven in ethylenediaminetetraacetic acid (EDTA) tubes. PCT determinations were made using a centralized time-resolved amplified cryptate emission technology-based assay (Kryptor® PCT, Thermo Scientific Biomarkers [B · R · A · H · M · S AG], Hennigsdorf, Germany) with a 0.06 μg/L functional sensitivity [19]. CRP concentrations were determined by an enzyme immunoassay having a detection limit <5 mg/dL (EMIT; Merck Diagnostica, Zurich, Switzerland). For the purpose of comparing cut-off ranges, PCT and CRP were stratified in four corresponding groups each, namely for PCT <0.1 μg/L, 0.1-0.25 μg/L, >0.25-0.5 μg/L and >0.5 μg/L, and for CRP ≤20 mg/dL, 21–50 mg/dL, >51-99 mg/dL, and ≥100 mg/dL, respectively, based on the use of these markers in previous antibiotic stewardship trials in the low acuity setting [2, 20, 21].

### Outcomes

In line with the initial study protocol [18], the predefined primary endpoint was the number of days, within the first 14 days after baseline, during which a patient’s daily activities (work or recreation) were restricted by a respiratory tract infection. The secondary endpoint was persistence of discomfort from infection at day 14. Both endpoints were assessed by seven medical students, blinded to the goal and design of the study, by conducting standardized follow-up interviews by telephone at 14 and 28 days after baseline.

### Statistical analysis

We used descriptive statistics including mean with standard deviation, median with interquartile range (IQR), and frequencies to describe the populations, as appropriate.

Assessment of correlation between biomarkers was performed by Spearman’s rank correlation analysis and visualized with scatter-plots. We looked at correlations between continuous biomarker levels and across cut-off ranges as defined above. For biomarker kinetics, we defined the change from baseline to day seven and classified the relative change into 4 groups (decrease of <25 %, 25–50 %, 50-75 % and >75 %).

To investigate associations between initial biomarker levels and endpoints, we used linear and logistic regression analysis. We adjusted for sociodemographic characteristics (age, gender, existence of comorbidities and education level) and in a second model also for disease severity (e.g. days spent with restricted activities before randomization and antibiotic prescription). Analyses were performed in the overall ARI patient population and in subgroups of different ARI types (upper ARI, non-pneumonic lower ARI, and pneumonic lower ARI). We calculated sensitivity and specificity using different clinically established cut-offs as defined above.

Statistical t-Tests were carried out at a 5 % significance level. All statistical analyses were performed with STATA Version 12.1 (Stata Corp, College Station, TX, USA).

## Results

### Patient population

This study included a total of 458 patients with a median age of 45 years (60 % females). Forty percent (184 patients) had upper ARI, particularly acute rhinosinusitis and acute pharyngitis/tonsillitis. Lower non-pneumonic ARI was diagnosed in 45 % of patients with acute bronchitis being the pre-dominant diagnosis (28 %) and 15 % had pneumonic lower RTI, namely CAP. Chest X-ray was performed in 121 patients, in 23 % of non-pneumonic ARI and in 97 % of CAP patients with non-pneumonic and pneumonic LRTI. The median reported degree of discomfort on recruitment was 6 – 7 (on a scale of 0 – 10 with 0 meaning no discomfort at all). Additional baseline characteristics of the study population are summarized in Table 1.

**Table 1:** Patient characteristics

	All patients (*n* = 458)
Demographics	
Age (years) – median [IQR]	45 (32–62)
Female gender – n (%)	273 (59.6 %)
Comorbidities	
Presence of any comorbidity – n (%)	70 (15.3 %)
Finale diagnosis	
Upper RTI	184 (40.2 %)
Acute rhinosinusitis – n (%)	104 (22.7 %)
Acute pharyngitis/tonsillitis – n (%)	75 (16.4 %)
Acute otitis media – n (%)	5 (1.1 %)
Lower non-pneumonic RTI	205 (44.8 %)
Common cold – n (%)	31 (6.8 %)
Acute bronchitis – *n* (%)	140 (30.6 %)
Influenza – *n* (%)	4 (0.9 %)
Exacerbated COPD – *n* (%)	21 (4.6 %)
Exacerbated asthma – *n* (%)	9 (2 %)
Lower pneumonic RTI	69 (15.1 %)
Medical outcome of patients	
Days with restricted activities within 14 days – median [IQR]	9 (6–12)
Persisting discomfort after 14 days	208 (45.7 %)
Degree of discomfort (scale 1–10) – median [IQR]	2 (1–5)
Biomarkers	
CRP mg/dL median (SD) [IQR]	31 (60) (8.3-72.7)
PCT μg/L median (SD) [IQR]	0.08 (2.11) (0.05-0.11)

### Correlation between PCT and CRP in different ARIs

**Fig. 1:**
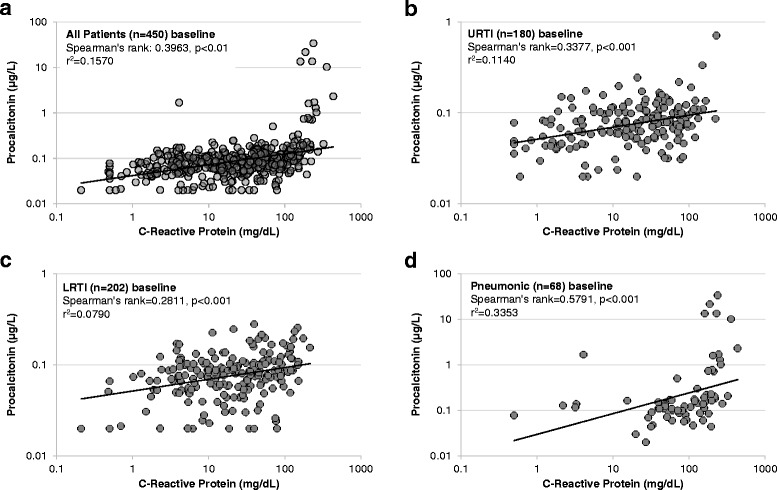
**a**-**d**: Correlation of Procalcitonin and CRP levels in lower and upper respiratory tract infections at baseline

**Fig. 2:**
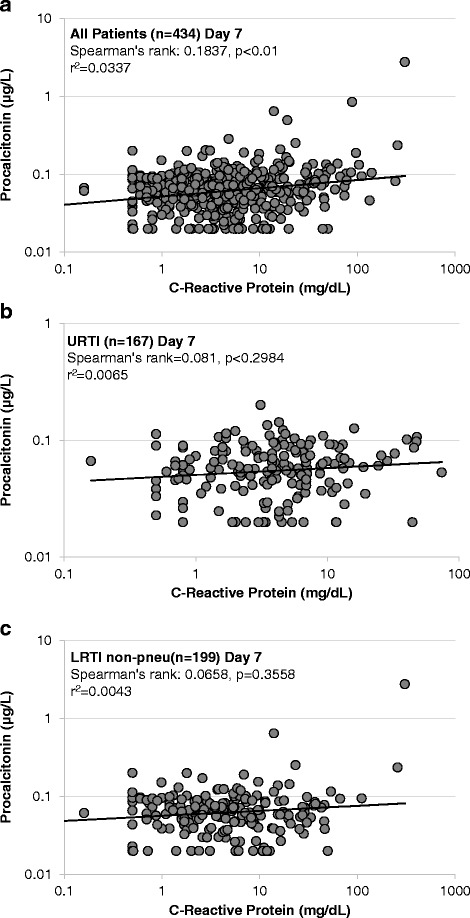
**a**-**c**: Correlation of Procalcitonin and CRP levels in lower and upper respiratory tract infections on day 7

Figures 1 and 2 show correlations of CRP and PCT in the overall population, and stratified by subgroups at baseline and on day seven. At baseline, we found significant correlations with however a low correlation coefficient between the two markers in the overall study population (r^2^ = 0.16), as well as in all subgroups. Highest correlations were found in the pneumonic lower ARI group (r^2^ = 0.34). On day seven, there were again weak correlations between both biomarkers in the overall population as well as in the subgroups.

We also investigated correlation of markers across well-established cut-off ranges (Tables 2 and 3). At baseline, 42 % of patients were in similar cut-off ranges with 9 % of patients being in higher PCT ranges and 49 % in higher CRP ranges. For day seven, 76 % of patients were in similar cut-off ranges with 13 % of patients being in higher PCT ranges and 11 % in higher CRP ranges. Looking at kinetics found 11 % of patients had a similar decrease in biomarker level with 75 % of patients having a stronger CRP decrease and 9 % a stronger PCT decrease. An increase in biomarker level was observed in 5 % of patients.

**Table 2:** Classification of biomarkers at baseline and day seven

Baseline		Overall (*n* = 450)			
	CRP < 20	CRP 20-50	CRP 50-100	CRP > 100	Total
PCT <0.1	141	82	62	15	300
	47 %	27.3 %	20.7 %	5 %	100 %
PCT0.1-0.25	38	34	25	33	130
	29.2 %	26.2 %	19.2 %	25.4 %	100 %
PCT 0.25-0.5	0	1	1	3	5
	0 %	20 %	20 %	60 %	100 %
PCT > 0.5	1	0	0	14	15
	6.7 %	0 %	0 %	93.3 %	100 %
Total	180	117	88	65	450
	40 %	26 %	19.6 %	14.4 %	100 %
Day 7		Overall (*n* = 434)			
	CRP < 20	CRP 20-50	CRP 50-100	CRP > 100	Total
PCT <0.1	322	30	5	3	360
	89.4 %	8.3 %	1.4 %	0.8 %	100 %
PCT0.1-0.25	52	7	6	3	68
	76.5 %	10.3 %	8.8 %	4.4 %	100 %
PCT 0.25-0.5	2	1	0	0	3
	66.7 %	33.3 %	0 %	0 %	100 %
PCT > 0.5	1	0	1	1	3
	33.3 %	0 %	33.3 %	33.3 %	100 %
Total	377	38	12	7	434
	86.9 %	8.8 %	2.8 %	1.6 %	100 %

**Table 3:** Classification of biomarkers at kinetics

Kinetics			Overall (*n* = 428)			
	Increase (CRP)	Decrease (CRP) 0 - 25 %	Decrease (CRP) 25 - 50 %	Decrease (CRP) 50-75 %	Decrease (CRP) > 75 %	Total
Increase (PCT)	22	14	17	26	53	132
	16.7 %	10.6 %	12.9 %	19.7 %	40.2 %	100 %
Decrease (PCT) 0–25 %	8	6	9	10	51	84
	9.5 %	7.1 %	10.7 %	11.9 %	60.7 %	100 %
Decrease (PCT) 25-50 %	6	5	7	17	60	95
	6.3 %	5.3 %	7.4 %	17.9 %	63.2 %	100 %
Decrease (PCT) 50–75 %	2	2	5	9	66	84
	2.4 %	2.4 %	6 %	10.7 %	78.6 %	100 %
Decrease (PCT) > 75 %	1	0	1	4	27	33
	3 %	0 %	3 %	12.1 %	81.8 %	100 %
Total	39	27	39	66	257	428
	9.1 %	6.3 %	9.1 %	15.4 %	60.1 %	100 %

### Association of admission biomarker levels and adverse outcome

Table 4 summarizes the results of regression analyses investigating the association of initial biomarker levels and adverse outcomes, namely days with restricted activities and persistence of ongoing discomfort at day 14. Analyses were performed according to baseline and day seven biomarker levels. For CRP, we found levels >100 mg/dL to be significantly associated with days of restricted activities (coefficient: 1.6 (95 % CI: 0.5, 2.6), *p* = 0.005). For day seven, results were similar with again levels >100 mg/dL being significantly associated in sociodemographically adjusted analysis (coefficient: 3.3 (95 % CI: 0.5, 6.2), *p* = 0.022) and a trend in fully adjusted analysis. For PCT, only initial levels >0.5ug/L were significantly associated with days with restricted activities in sociodemographically adjusted analysis (coefficient 2.0, 95 % CI 0.0 to 4.0) with a trend after full adjustment (*p* = 0.089).

**Table 4:** Biomarker at baseline and day seven as predictor for days with restricted activities or persistence of discomfort after 14 days

Days with restricted activites: coefficient (95 % CI)						
		*Demographic*-*adjusted model*	*Fully adjusted model* ^a^		*Demographic*-*adjusted model*	*Adjusted* ^a^
Biomarker at baseline	CRP (mg/dL)	0.4 (−0.2 to 0.9), *p* = 0.169	0.3 (−0.3 to 0.9), *p* = 0.324	Biomarker on day 7	0.7 (0.1 to 1.4), *p* = 0.035	0.6 (−0.1 to 1.3), *p* = 0.075
	CRP ≤20	Reference group	Reference group		Reference group	Reference group
	CRP 20-50	0.4 (−0.5 to 1.3), *p* = 0.37	0.4 (−0.5 to 1.3), *p* = 0.407		0.7 (−0.6 to 2.0), *p* = 0.264	0.8 (−0.5 to 2.1), *p* = 0.252
	CRP 50-100	0.0 (−1.0 to 1.0), *p* = 0.956	−0.1 (−1.0 to 0.9), *p* = 0.920		2.5 (0.3 to 4.7), *p* = 0.024	2.4 (0.2 to 4.5), *p* = 0.035
	CRP >100	1.6 (0.5 to 2.6), *p* = 0.005	1.4 (0.3 to 2.5), *p* = 0.014		3.3 (0.5 to 6.2), *p* = 0.022	2.7 (−0.3 to 5.7), *p* = 0.078
	PCT (μg/L)	0.9 (0 to 1.9), *p* = 0.048	0.8 (−0.2 to 1.8), *p* = 0.123		0.7 (−0.6 to 2.1), *p* = 0.297	0.5 (−0.9 to 1.9), *p* = 0.484
	PCT ≤0.1	Reference group	Reference group		Reference group	Reference group
	PCT 0.1-0.25	0.4 (−0.4 to 1.2), *p* = 0.320	0.3 (−0.5 to 1.1), *p* = 0.407		0.6 (−0.4 to 1.6), *p* = 0.249	0.5 (−0.5 to 1.5), *p* = 0.353
	PCT 0.25-0.5	1.6 (−1.3 to 4.4), *p* = 0.288	1.2 (−1.8 to 4.1), *p* = 0.434		2.9 (−1.5 to 7.3), *p* = 0.194	2.3 (−2.1 to 6.7), *p* = 0.300
	PCT >0.5	2.0 (0.0 to 4.0), *p* = 0.045	1.8 (−0.3 to 3.8), *p* = 0.089		−0.3 (−4.6 to 4.1), *p* = 0.900	−0.7 (−5.1 to 3.7), *p* = 0.751
Persistence of discomfort after 14 days: coefficient (95 % CI)						
		*Demographic*-*adjusted model*	*Adjusted* ^a^		*Demographic*-*adjusted model*	*Adjusted* ^a^
Biomarker at baseline	CRP (mg/dL)	1.0 (0.8 to 1.4), *p* = 0.831	1.0 (0.8 to 1.4), *p* = 0.828	Biomarker on day 7	1.7 (1.2 to 2.4), *p* = 0.005	1.7 (1.2 to 2.5), *p* = 0.005
	CRP ≤20	Reference group	Reference group		Reference group	Reference group
	CRP 20-50	1.2 (0.7 to 1.9), *p* = 0.517	1.2 (0.7 to 2), *p* = 0.436		1.6 (0.8 to 3.2), *p* = 0.203	1.7 (0.8 to 3.4), *p* = 0.166
	CRP 50-100	1.0 (0.6 to 1.8), *p* = 0.862	1.1 (0.6 to 1.8), *p* = 0.828		3.4 (0.9 to 13.1), *p* = 0.078	3.4 (0.8 to 13.5), *p* = 0.085
	CRP >100	1.2 (0.7 to 2.2), *p* = 0.477	1.2 (0.7 to 2.3), *p* = 0.514		6.4 (0.7 to 54.8), *p* = 0.091	4.5 (0.5 to 40.9), *p* = 0.18
	PCT (μg/L)	1.1 (0.6 to 1.7), *p* = 0.845	1.0 (0.6 to 1.7), *p* = 0.939		0.9 (0.4 to 1.9), *p* = 0.804	0.8 (0.4 to 1.7), *p* = 0.588
	PCT ≤0.1	Reference group	Reference group		Reference group	Reference group
	PCT 0.1-0.25	0.8 (0.5 to 1.3), *p* = 0.439	0.8 (0.5 to 1.3), *p* = 0.466		1.1 (0.7 to 1.9), *p* = 0.655	1.1 (0.6 to 1.9), *p* = 0.738
	PCT 0.25-0.5	3.3 (0.6 to 18.0), *p* = 0.166	2.9 (0.5 to 16.3), *p* = 0.229		2.5 (0.2 to 28.5), *p* = 0.460	1.8 (0.2 to 21.4), *p* = 0.63
	PCT >0.5	1.6 (0.5 to 4.7), *p* = 0.402	1.5 (0.5 to 4.5), *p* = 0.486		0.5 (0.0 to 5.7), *p* = 0.540	0.3 (0.0 to 4.6), *p* = 0.42

Adjusted for: gender, comorbidities, years of education, randomization (^a^additional adjustment: prescription of antibiotics, days with RA prior to randomization)

We also repeated the same analysis in the different subgroups, i.e. in patients with upper ARI and in lower pneumonic and non-pneumonic ARI. While for the upper ARI no significant associations were found, best results were seen in the non-pneumonic lower ARI group (see Additional files 1, 2, 3, 4 and 5).

Finally, we also investigated associations of biomarkers with persistent discomfort after 14 days. For this endpoint, no significant associations were found for both markers at baseline. For the day seven analysis, significant results were found for CRP, but not PCT.

## Discussion

The findings of this analysis including a large primary care population with different types of ARI are threefold. First, correlations between CRP and PCT levels were low in the overall population and particularly in the upper and non-pneumonic lower ARI. Second, high levels of both markers in the highest cut-off range were associated with days with restricted activity but not with ongoing discomfort. Third, kinetics did not significantly improve outcome prediction.

To date, ARI still represent an important public health issue affecting millions of patients around the world. Incorrect or delayed diagnosis may directly lead to severe complications and increases morbidity and mortality, especially in the inpatient setting [3]. Sputum and blood cultures are commonly seen as the gold standard. Still, these techniques have low sensitivity and lack practicability (e.g. in case of non-productive cough) [22, 23]. Because differentiation of mild viral infection from more severe infections is challenging, clinicians often start empiric antibiotic treatment which increases resistance of common bacteria and causes drug-related side effects [24, 25]. In this context, objectively measurable blood biomarkers may help to assess severity and need for more intensive treatment. There is still uncertainty about optimal use of PCT or CRP in the primary care setting for the management of patients. Herein, our analysis including a large and well defined cohort of ARI patients sheds new light on this important topic.

Compared to higher acuity settings, patients with low acuity ARI had low PCT levels with minimal changes over seven days. Yet, CRP levels showed a higher variability. This is also consistent with findings from other primary care studies [26], suggesting that PCT is more specific to bacterial infections and low in patients with mainly viral infections, while CRP increases independent of infection type as a “inflammatory marker”. Interestingly, as shown in our correlation analyses, patients displayed differences in biomarker profiles with low correlation between markers and between cut-off ranges. Because both markers had suboptimal prognostic accuracy for outcome prediction, and because no goldstandard for ARI in need of antibiotics exist, only a head to head trial comparing the performance of both markers in regard to antibiotic management may help to clarify the question which marker is more helpful in the management of ARI patients.

Several limitations should be considered when interpreting our results. First, this is a secondary analysis of previous randomized study with a different study question, i.e., whether antibiotic therapy guided by PCT reduces the use of antibiotics without increasing the restrictions experienced by patients. Second, no external validation of ARI diagnoses was done. Instead, physicians were encouraged to use updated guidelines presented in an interactive seminar. Also, chest X-ray was not mandatory for diagnosis of CAP and some patients may have been misclassified. In addition, we classified patients with common cold, influenza, and acute laryngitis-bronchitis as lower ARI because they frequently presented with cough as a main symptom and pathological findings in chest auscultation. Third, we focused on adverse outcomes as specified in the original trials but did not look into other adverse outcomes such as unplanned readmission to the GP, hospital admission, and respiratory failure among others. Further, PCT was not measured on site, due to lack of sensitive POC at that time. Future studies comparing these markers should also look into POC system for both markers in primary care.

## Conclusion

This is the first large and comprehensive study investigating correlations between CRP and PCT and their predictive value in the primary care setting in patients with different types of ARI. The low correlations between the two biomarkers and the only moderate prognostic accuracy calls for a head-to-head trial comparing the ability of both markers to manage primary care patients with ARI to answer the question which marker is superior.

### Ethics approval and consent to participate

The ethics committee of the University Hospital Basel, Switzerland, approved the trial protocol. The trial was supervised by an independent monitoring board consisting of a general internist in primary care, an infectious disease specialist, and a pneumologist. All participating physicians and patients gave written informed consent.

### Consent for publication

Not applicable.

### Availability of data and materials

Due to our local Institutional review board policy, we do not have permission to make the data sets on which the conclusions of the paper rely publicly available. A truncated data set (removing all potentially identifying features) may be made available on an individual request basis.

## Additional files

Additional file 1: Subgroup classification at baseline. (PPTX 128 kb) Additional file 2: Subgroup classification on day seven. (PPTX 89 kb) Additional file 3: Biomarker at baseline and day seven as predictors for days with restricted activities or persistence of discomfort after 14 days according to subgroups. URTI. (PPTX 86 kb) Additional file 4: Biomarker at baseline and day seven as predictors for days with restricted activities or persistence of discomfort after 14 days according to subgroups. LRTI non-pneumonic. (PPTX 86 kb) Additional file 5: Biomarker at baseline and day seven as predictors for days with restricted activities or persistence of discomfort after 14 days according to subgroups. LRTI pneumonic. (PPTX 86 kb)

## Abbreviations

ARTI: acute respiratory tract infection
CAP: community acquired pneumonia
CRP: C-reactive protein
GP: general practitioner
LRTI: lower respiratory tract infection
PCT: procalcitonin
POC (T): point of care (test)
RTI: respiratory tract infection
PARTI: procalcitonin-guided antibiotic use versus a standard approach for acute respiratory tract infections in primary care
