# Supplementary figures and images for: Infection biomarkers in primary care patients with acute respiratory tract infections–comparison of Procalcitonin and C-reactive protein
Source: BMC Pulm Med. 2016 Mar 24;16:43. doi: 10.1186/s12890-016-0206-4 (PMC4806430; doi:10.1186/s12890-016-0206-4)

## Slide 1
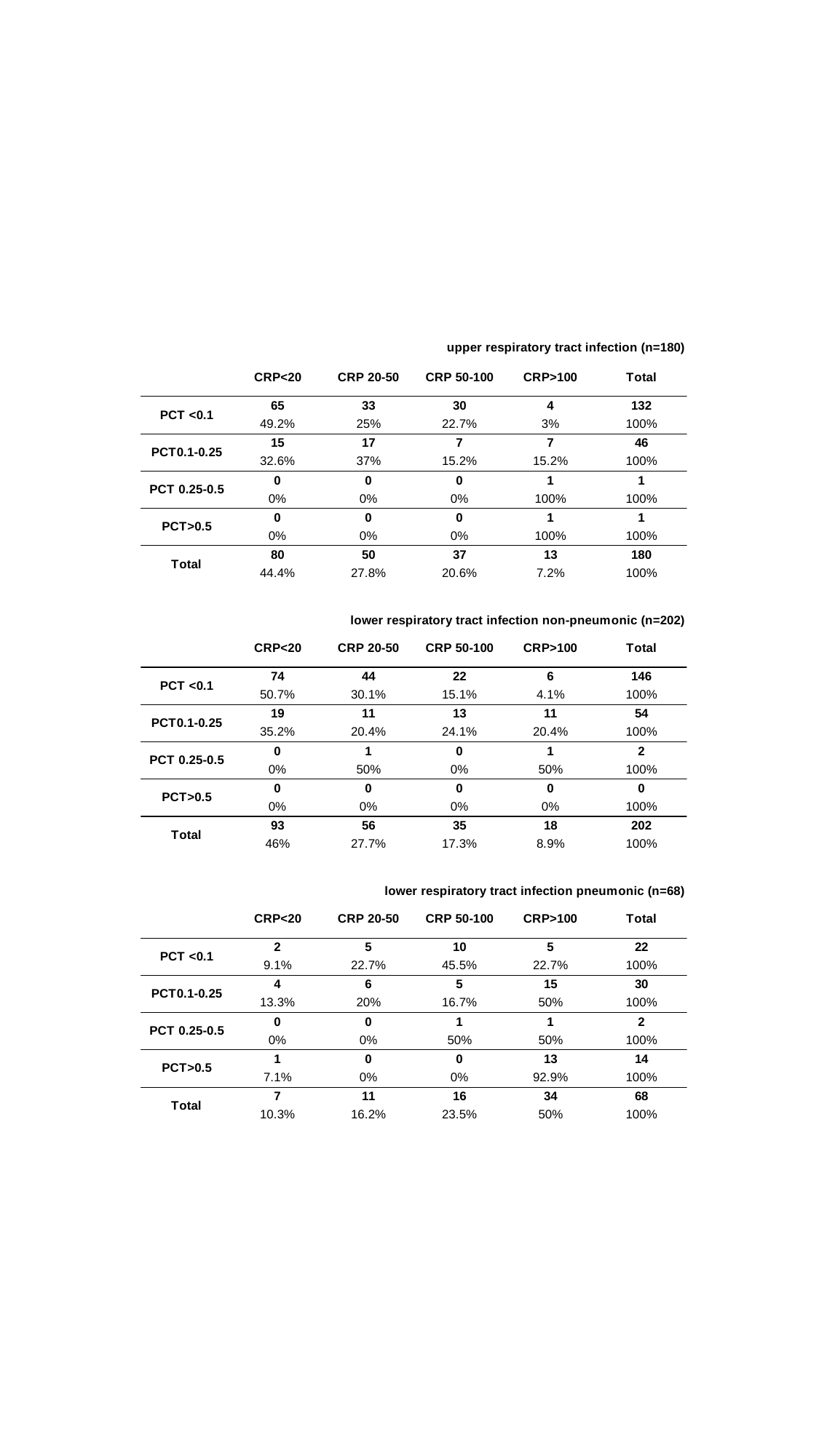

Supplement: Additional file 1: — Subgroup classification at baseline. (PPTX 128 kb) [file 12890_2016_206_MOESM1_ESM.pptx]

## Slide 1
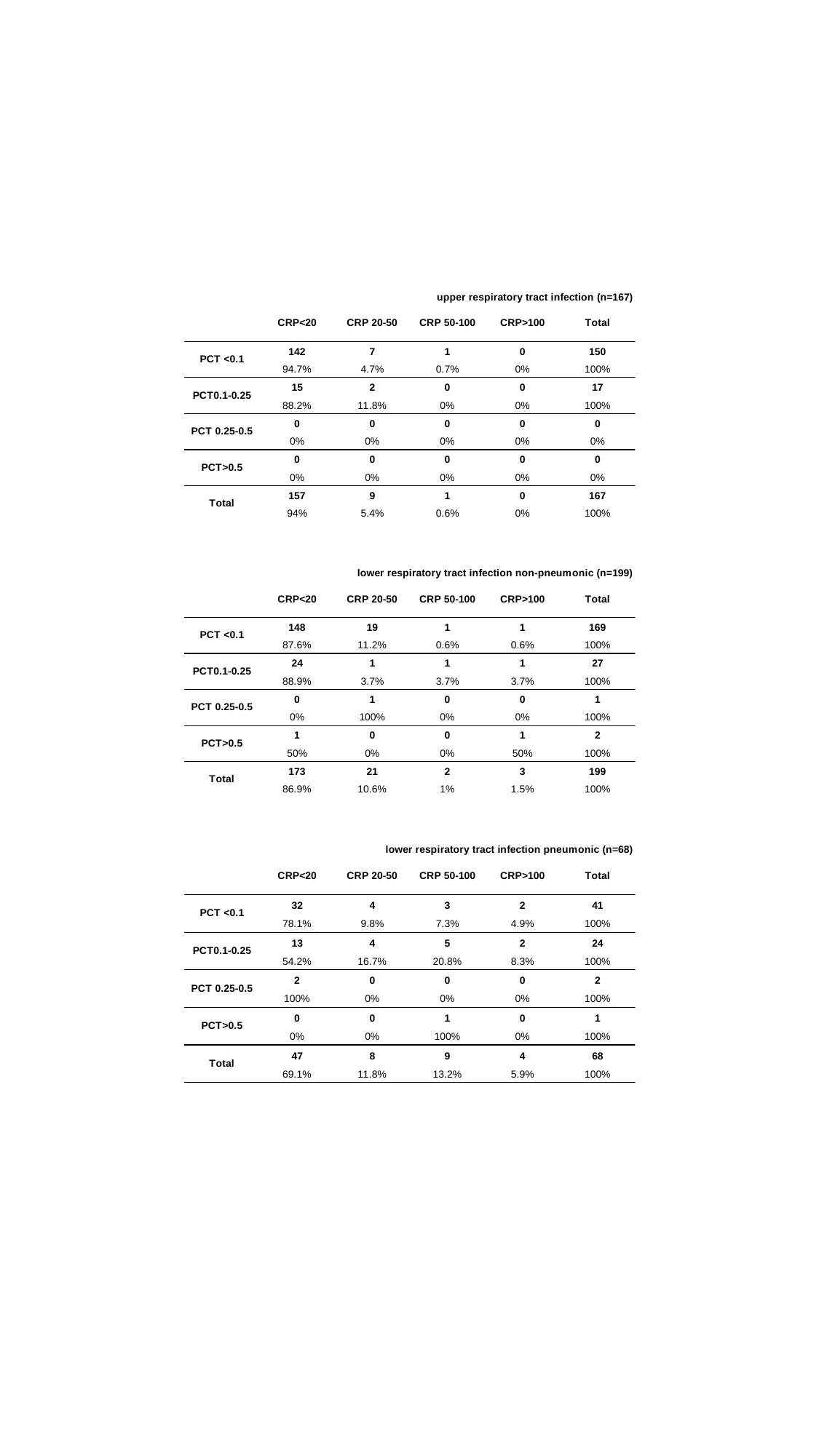

Supplement: Additional file 2: — Subgroup classification on day seven. (PPTX 89 kb) [file 12890_2016_206_MOESM2_ESM.pptx]

## Slide 1
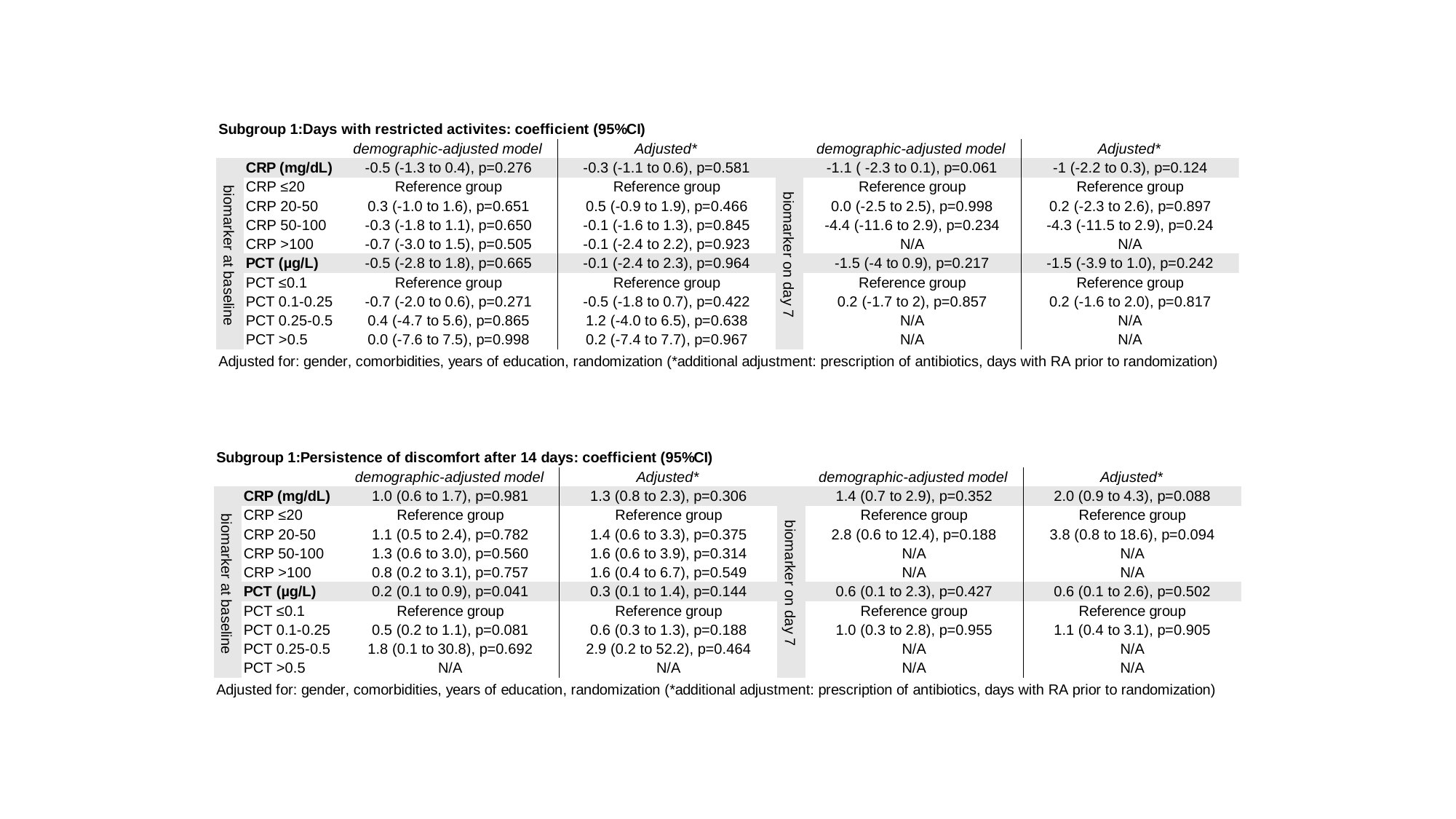

Supplement: Additional file 3: — Biomarker at baseline and day seven as predictors for days with restricted activities or persistence of discomfort after 14 days according to subgroups. URTI. (PPTX 86 kb) [file 12890_2016_206_MOESM3_ESM.pptx]

## Slide 1
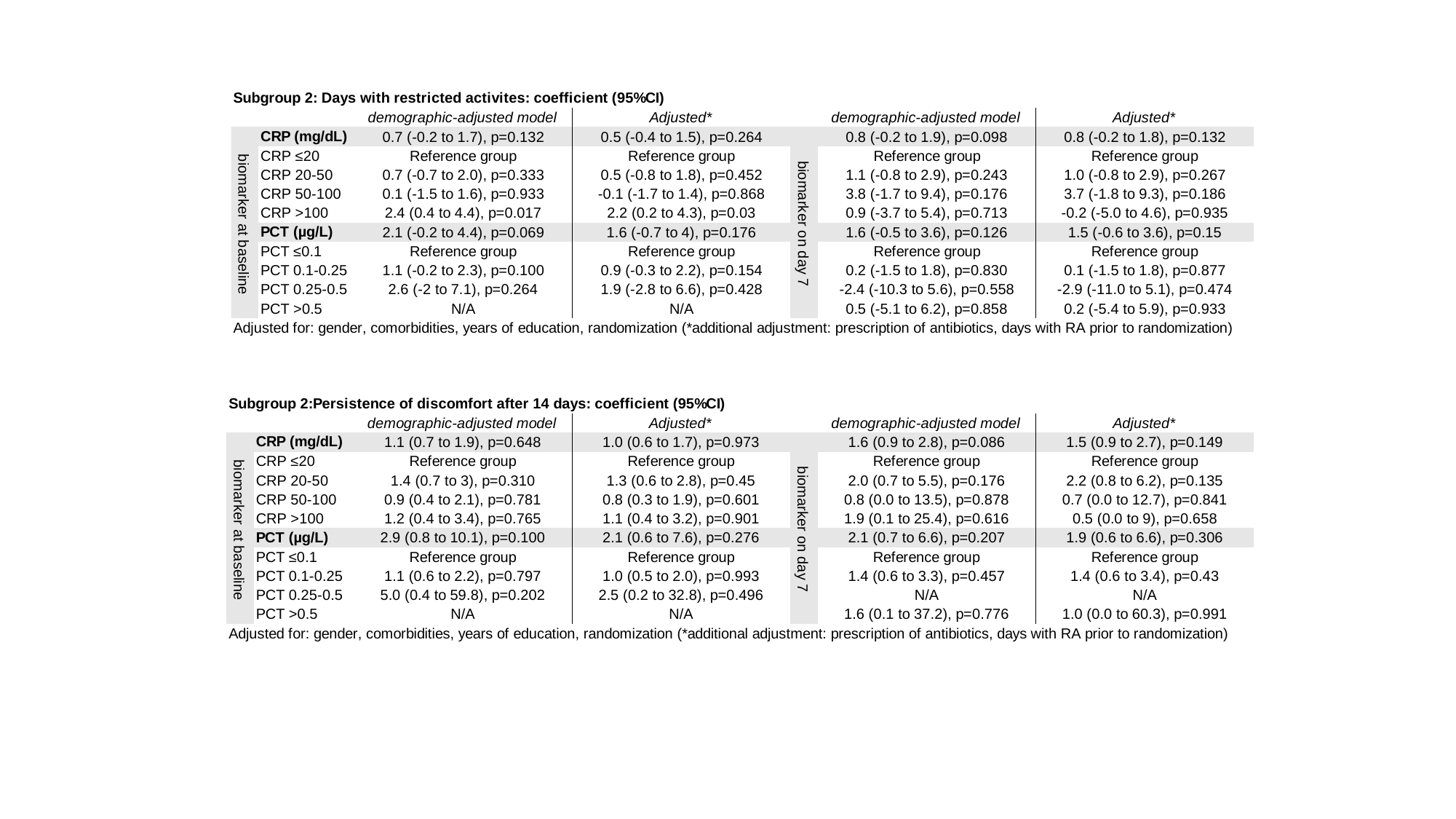

Supplement: Additional file 4: — Biomarker at baseline and day seven as predictors for days with restricted activities or persistence of discomfort after 14 days according to subgroups. LRTI non-pneumonic. (PPTX 86 kb) [file 12890_2016_206_MOESM4_ESM.pptx]

## Slide 1
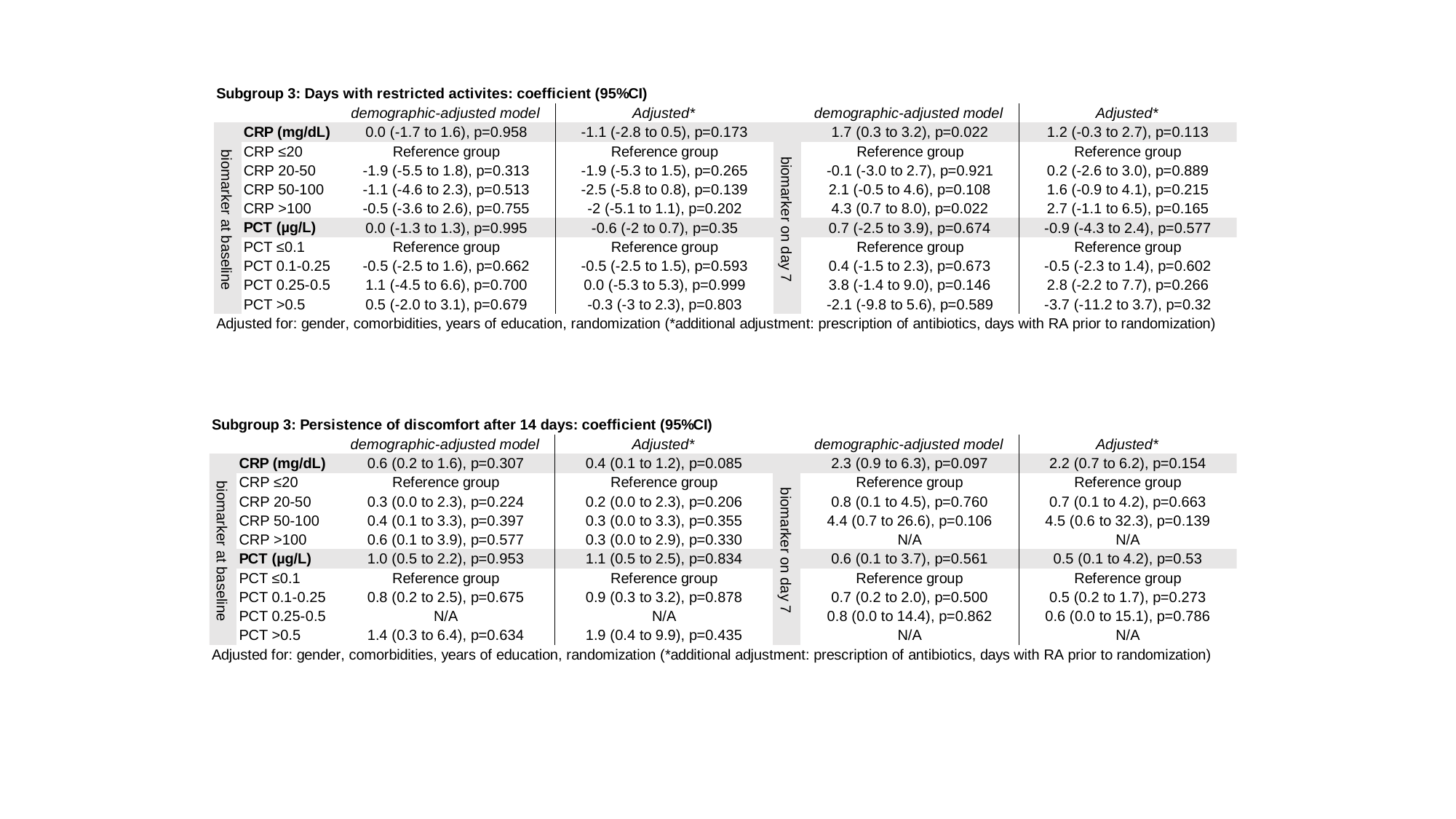

Supplement: Additional file 5: — Biomarker at baseline and day seven as predictors for days with restricted activities or persistence of discomfort after 14 days according to subgroups. LRTI pneumonic. (PPTX 86 kb) [file 12890_2016_206_MOESM5_ESM.pptx]
